# Anterior Quadratus Lumborum Block: A Regional Anaesthetic Technique for the Management of Acute Back Pain in the Emergency Department

**DOI:** 10.7759/cureus.96504

**Published:** 2025-11-10

**Authors:** Janam Vadgama, Samia Ahmad, Athmaja Thottungal, Salman Naeem

**Affiliations:** 1 Medicine, King's College Hospital, London, GBR; 2 General Practice, Hill Cove Medical Centre, Adelaide, AUS; 3 Anaesthesiology, East Kent Hospitals University NHS Foundation Trust, Canterbury, GBR; 4 Emergency Department, Flinders Medical Centre, Adelaide, AUS; 5 College of Medicine and Public Health, Flinders University, Adelaide, AUS

**Keywords:** acute lower back pain, anterior quadratus lumborum block, emergency medicine, quadratus lumborum syndrome, regional anaesthesia

## Abstract

Acute lower back pain presents a major clinical and economic burden, accounting for substantial emergency department (ED) attendances and significant healthcare utilisation. Quadratus lumborum (QL) syndrome is an under-recognised cause of acute lower back pain, often resistant to standard pharmacological therapy. While ultrasound-guided anterior quadratus lumborum block (AQLB) has demonstrated analgesic efficacy in perioperative settings, its role as a rescue analgesic in the ED remains underexplored. A 49-year-old male presented to the ED with severe bilateral flank pain (numeric rating scale (NRS) = 8/10), radiating to the left leg and groin. This was his second ED presentation in one week. Imaging showed no renal calculus, and laboratory results were largely unremarkable except for mild leukocytosis and chronic kidney disease stage 3. His pain was refractory to multimodal systemic analgesia, including opioids and non-steroidal anti-inflammatory drugs. On review, he was found to have bilateral QL tenderness limiting mobility. Given ongoing severe pain (NRS = 8/10), ultrasound-guided bilateral AQLB was performed using 30 mL of 0.18% ropivacaine on each side with a 21G SonoPlex®II needle (Pajunk®, Geisingen, Germany), accompanied by intravenous dexamethasone (6 mg). Pain scores improved to 2/10 NRS within 30 minutes, allowing mobilisation and discharge within 24 hours on a simple analgesia regimen and physiotherapy advice. Pain recurrence occurred three days later, prompting re-presentation. This case highlights AQLB as a feasible and effective rescue technique for acute back pain in the ED, particularly in patients with QL syndrome who do not respond adequately to systemic analgesics. The rapid and substantial pain reduction post-block suggests a significant role for AQLB in interrupting QL pain-spasm cycles, enabling early mobilisation and reducing hospital admissions. Although systemic analgesia may have contributed, the temporal relationship supports the block’s primary effect. Adjuncts such as dexamethasone and gabapentinoids may prolong block duration, though evidence in AQLB for non-operative pain remains limited. Broader adoption of regional anaesthesia in ED practice could improve patient outcomes, decrease opioid use, and optimise healthcare resource utilisation. Future research should focus on standardising the technique, evaluating the duration of effect, and assessing long-term outcomes, including mobility, functional recovery, and healthcare utilisation. This case suggests that AQLB is a feasible and effective opioid-sparing rescue technique for the management of acute back pain in the ED secondary to QL syndrome. It adds to emerging evidence supporting its role as a safe, effective, and resource-efficient intervention, meriting further prospective evaluation.

## Introduction

Acute back pain, defined as back pain lasting less than four weeks [1], represents a significant health burden, accounting for approximately 3-4% of emergency department (ED) attendances [2]. It is a major cause of disability, functional limitation, and work absence, with frequent recurrences leading to substantial healthcare utilisation. The financial impact is considerable in the UK, with costs of £32.5 million in 2015 (£35.9 million in 2020), extrapolating to £3.2 billion (£3.5 billion in 2020) nationwide in primary care alone [3]. Most cases are mechanical in origin (97%), typically involving muscular, ligamentous, or joint strain, while less common causes include trauma, degenerative disease, malignancy, infection, inflammation, metabolic disorders, referred pain, congenital abnormalities, and psychiatric illness [4].

Acute non-traumatic lower back pain mostly involves the paraspinal muscles, i.e., erector spinae and quadratus lumborum (QL) group of muscles. The QL muscle is a frequently overlooked source of acute lower back pain. Located in the posterolateral lumbar region, it attaches to the iliac crest, transverse processes of the lumbar vertebrae, and the inferior border of the 12th rib [5]. Functionally, it contributes to spinal stabilisation, lumbar extension, and lateral flexion [5]. Dysfunction arises when compensatory overuse leads to stiffness and spasm, producing quadratus lumborum pain syndrome, a form of myofascial pain [6]. The pain is typically dull, aching, and radiates from the back to the hip and groin. On examination, patients may exhibit tenderness over the QL muscle, restricted lumbar motion, and pain reproduced by resisted lateral flexion. The resulting pain perpetuates a cycle of spasm and further pain [1], underscoring the importance of timely and effective treatment. Acute treatment involves systemic analgesics and physical therapy, including stretching exercises of the QL muscle and strengthening of the core and gluteal muscles.

Globally, 9.6% of ED presentations with acute lower back pain result in hospital admission [7] and 30% of patients require multiple visits [8], imposing a considerable burden on healthcare resources. This underscores the need for improved treatment strategies that provide effective analgesia, facilitate early mobilisation, and reduce recurrent admissions. First-line pharmacological management of acute back pain typically includes paracetamol, non-steroidal anti-inflammatory drugs (NSAIDs), and opioids [9]. While effective for short-term relief, these agents are limited by side effects, contraindications, and risks of overuse [2]. Ultrasound-guided regional anaesthesia (USGRA) has emerged as a promising option, offering minimally invasive, cost-effective analgesia [1].

Anterior quadratus lumborum block (AQLB) targets the fascial plane between the quadratus lumborum and psoas major, with potential spread to the lumbar plexus, covering the T7 to L4 dermatomes [10]. Originally applied in perioperative care [11], various QL blocks have demonstrated efficacy in systematic reviews, reducing pain scores for up to 48 hours and delaying rescue analgesia [11]. More recently, case reports suggest AQLB's benefits in the ED settings, including significant reductions in the Visual Analogue Scale (VAS) and Oswestry Disability Index (ODI) scores [1], alongside improved mobilisation and engagement in multimodal therapies such as physiotherapy, which may improve clinical outcomes and reduce the chronicity of back pain. However, evidence remains limited with variability in technique, injectate, and follow-up [12], with some studies reporting no advantage over multimodal analgesia alone [13]. This case report adds to the growing evidence by detailing its application in acute back pain, specifically QL syndrome, including technique, outcomes, and safety profile.

## Case presentation

A 49-year-old male presented to the ED with bilateral flank pain of 8/10 on the numeric rating scale (NRS) [14], which had been ongoing for a few months but had been exacerbated the night before. His pain was severe and crampy in nature, radiating from his back to his left leg and groin, relieved by sitting in a hunched position, and worsened by movement and raising his leg. He reported associated chills and one episode of vomiting containing gastric contents. This was his second presentation to the ED within a week with similar back pain. He had an ultrasound scan of the kidney, ureters, and bladder (KUB), showing a non-obstructive stone in the lower pole of the left kidney during the last admission and was subsequently discharged home with a diagnosis of left renal colic.

The patient had a past medical history of right recurrent renal calculi, right inguinal hernia repair, hypertension, and stage 3 chronic kidney disease (CKD). He was a smoker but denied the use of alcohol and recreational drugs. He was also suffering from ongoing intermittent haematuria without any lower urinary tract symptoms (LUTS), which was being investigated for nephritis. He was taking the following medications regularly: amlodipine 10 mg once daily, buprenorphine 15 mcg per hour every week, candesartan 16 mg once daily, esomeprazole 40 mg once daily, tapentadol continuous release 50 mg once daily, and prednisolone 40 mg on a tapering dose.

**Figure 1:**
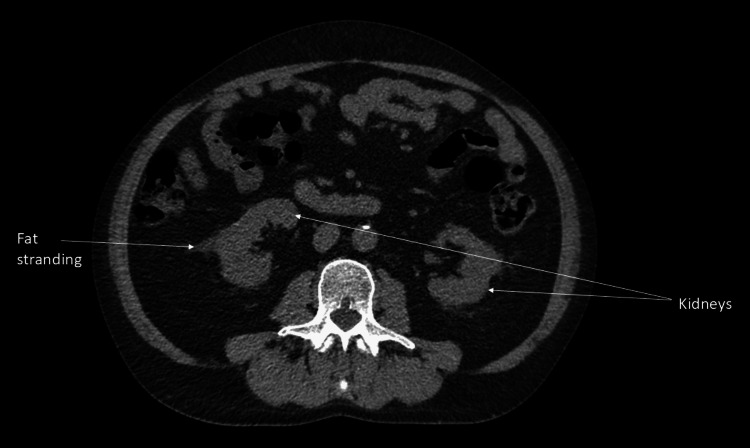
Computed tomography scan of the kidney, ureter, and bladder. Axial view of a non-contrast computed tomography scan of the abdomen demonstrating both kidneys. There are hyper-densities around the kidneys, as highlighted by the arrow, which are due to inflammation of the fat surrounding the kidneys.

On examination, his vital signs were as follows: respiratory rate of 18 per minute, saturating at 98% on air, heart rate of 82 beats per minute, temperature of 36.5°C, and blood pressure of 158/103 mmHg. He had warm, well-perfused peripheries with normal heart and lung sounds. His abdomen was soft; however, there was tenderness in the flanks bilaterally, which was worse on the left. Bowel sounds were present on auscultation. After initial investigation for left ureteric colic, as described in Table 1, a computed tomography scan of his kidney, ureter, and bladder was performed, which did not demonstrate a calculus in the kidney with bilateral fat standing around the kidneys showing long-standing nephritis, as shown in Figure 1.

**Table 1:** Blood investigations. INR: international normalised ratio; APTT: activated partial thromboplastin time.

Blood tests	Patient’s values	Reference range
Haemoglobin	143 g/L	135-175 g/L
Platelets	255 x 10^9^/L	150-450 x 10^9^/L
White blood cells	15.44 x 10^9^/L	4-11 x 10^9^/L
Sodium	140 mmol/L	135-145 mmol/L
Potassium	5.0 mmol/L	3.5-5.2 mmol/L
Creatinine	147 µmol/L	60-110 µmol/L
C-reactive protein	0.7 mg/L	0.0-8 mg/L
Albumin	39 g/L	34-48 g/L
Bilirubin	4 µmol/L	2-24 µmol/L
INR	0.8	0.9-1.2
APTT	27 U/L	21-38 U/L
Glucose	6.0 mmol/L	3.2-5.5 mmol/L

**Figure 2:**
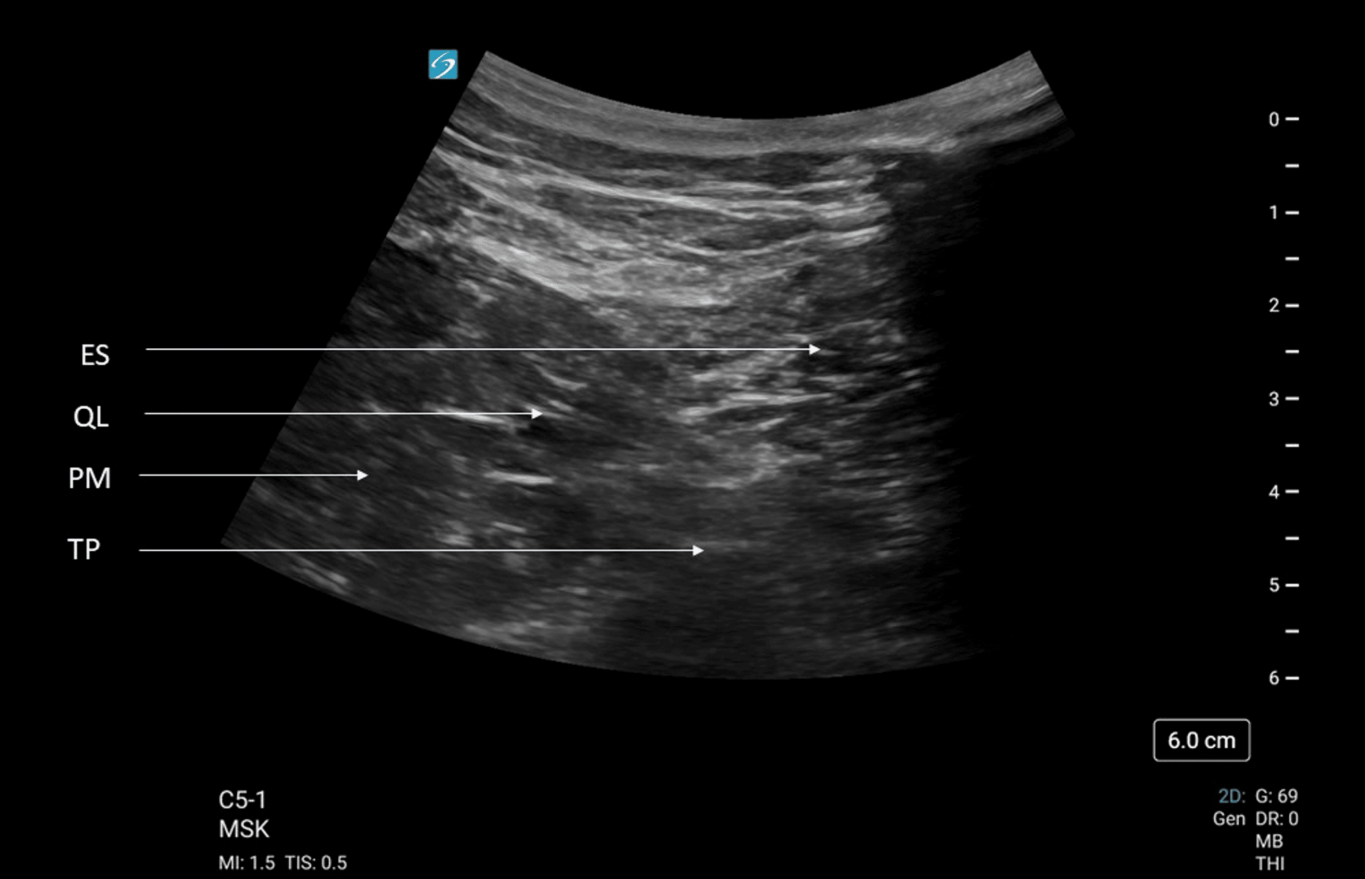
Oblique view of the right flank. Oblique view of the right flank showing spread of anechoic local anaesthetic (LA) in the fascial plane between psoas major (PM) and quadratus lumborum (QL). Erector spinae (ES) is posterior to QL. TP: transverse process.

**Figure 3:**
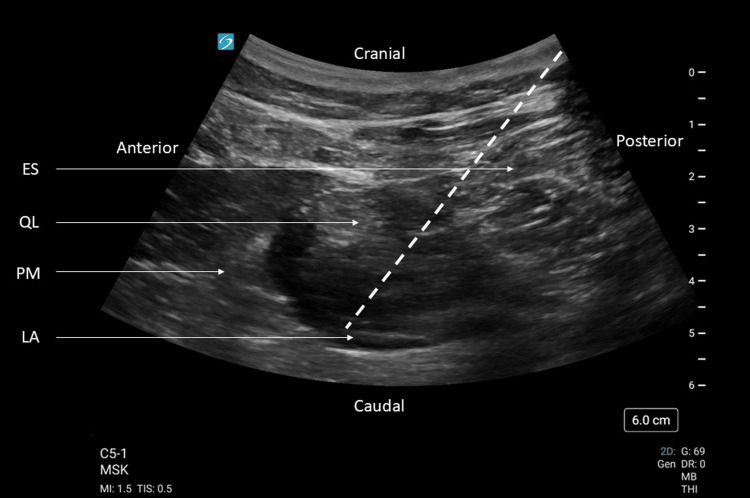
Anterior quadratus lumborum block. Oblique view of the right flank showing spread of anechoic local anaesthetic (LA) in the fascial plane between psoas major (PM) and quadratus lumborum (QL). Erector spinae (ES) is posterior to QL. The dotted line shows the trajectory of the needle for the block.

He was given fentanyl 50 mcg, oxycodone 10 mg, paracetamol 1 gm, and indomethacin 50 mg for his pain management; however, his pain remained at 6-8/10 on NRS despite analgesics. He was admitted to the short-stay unit for pain management. Upon review, he had pain on movement of his back, with tenderness over the lumbar erector spinae (ES) and bilateral QL. The lateral flexion test was positive bilaterally. Tenderness was more pronounced over the left QL. His pain was 8/10 on NRS eight hours post admission. He was consented for AQLB bilaterally as a rescue analgesic for refractory acute on chronic back pain. AQLB was performed on each side. A curvilinear probe was used with the musculoskeletal preset. The probe was placed just above the iliac crest of the patient in the axial plane, with the probe pointer pointing to the front of the patient. QL muscle was located between ES posteriorly, psoas major anteriorly, and the transverse process of the lumbar vertebra inferiorly to it. AQLB was performed with 30 ml of 0.18% ropivacaine using a 21G 100 mm SonoPlex®II facet tip needle (Pajunk®, Geisingen, Germany) under ultrasound guidance. Successful blocks were done bilaterally, and 6 mg of intravenous dexamethasone was given concurrently, as shown in Figures 2, 3.

He reported a pain score of 5/10 on NRS during the block, which reduced to an NRS score of 2/10 30 minutes post-block. He was able to mobilise and was discharged on paracetamol 1 gm four times per day, naproxen 250 mg thrice daily, and oxycodone 5 mg as required for breakthrough pain. He was advised about QL stretch exercises and physiotherapist follow-up. He reported a pain score of 2/10 on NRS 24 hours post discharge. However, his pain gradually got worse again three days after discharge, and he presented to the ED.

## Discussion

This case report highlights the potential of the AQLB as an effective rescue treatment for acute back pain management in the ED. In this patient, the block produced rapid and substantial analgesia, enabling mobilisation and discharge. However, pain recurrence within days underscores the need for comprehension of QL syndrome as a cause of acute lower back pain, the potential for AQLB as a rescue analgesic in patients with maximal systemic analgesia, QL pain-spasm cycles, and strategies to prolong block efficacy. Our patient had a recurrence of pain as he failed to engage with stretch exercises and physiotherapy. This will allow the successful treatment of QL syndrome-associated lower back pain, reduce systemic analgesic requirements, improve mobility, and reduce both hospital admissions and healthcare strain.

Myofascial pain secondary to QL syndrome is a considerable musculoskeletal pathology in patients reporting acute lower back pain [1]. Due to anatomical functions of the QL muscle, including spinal stabilisation in flexion, antagonism of trunk extension, and lateral flexion, strain often occurs, causing profound pain-spasm cycles [1]. Although complex musculoskeletal and neural pathways underpinning central sensitisation in QL syndrome are apparent [6], they are currently underdeveloped and require comprehensive evaluation to effectively utilise treatments such as AQLB.

Despite receiving maximal, multimodal systemic analgesia as a first-line management for acute lower back pain, many patients, including ours, have unresolved pain, thus paving the way for AQLB utilisation as a rescue therapy. Post-operatively, AQLB has provided superior analgesic results compared to opioid therapies [15], without adverse side effects including tolerance, nausea and vomiting, as well as reducing opioid requirements and length to first analgesic request [16]. However, an important consideration is the temporal association between block administration and pain relief. Although systemic analgesic agents were administered before and after the block, the immediate and substantial reduction in pain (NRS reduction of six points pre- and post block) strongly suggests a key role for AQLB in this patient’s improvement. However, separating the relative contributions of block and pharmacotherapy remains a challenge, specifically in a single-patient study, whereby further investigation into the underlying pathophysiology of QL spasm and its responsiveness to regional techniques is warranted.

Adjuncts such as dexamethasone and gabapentinoids have shown potential to prolong block duration. Dexamethasone increased median block duration by 37% when combined with ropivacaine 0.5% in a retrospective analysis of 1,040 patients [17] and extended analgesia by 488 minutes in a meta-analysis of 29 trials with long-acting local anaesthetics [18]. While large-scale data for AQLB are limited, this evidence suggests potential applicability, warranting further investigation to establish efficacy. Similarly, gabapentinoids may reduce central sensitisation to sustain analgesic effects. However, as AQLB-related pain may follow proposed pain-spasm cycles compared to distinct perioperative nociceptive pathways [16], the direct translatability of gabapentinoid efficacy remains uncertain, highlighting the need for targeted studies.

The broader implications of regional anaesthesia in the ED extend beyond immediate analgesia. In our case, AQLB provided rapid and effective pain control, allowing the patient to mobilise and be discharged within 24 hours of block administration. This observation is consistent with previous studies that have demonstrated statistically and clinically significant reductions in pain scores following hip arthroplasty at 12, 24, and 48 hours [19]. Early mobilisation further allows engagement in physiotherapy programmes, with a systematic review demonstrating that early physical therapy within 30 days of acute lower back pain reduces future healthcare service utilisation, including significant opioid use, spinal injections, and surgery [20]. Future studies could therefore evaluate not only pain scores, but mobility, discharge readiness, long-term functional recovery, and healthcare utilisation, to provide a holistic assessment of AQLB efficacy. In this way, the adoption of AQLB in acute care may not only improve patient outcomes but also optimise efficiency across emergency services.

## Conclusions

This case highlights QL syndrome as a cause of acute back pain. It also demonstrates the feasibility of AQLB as a regional technique in the ED for acute back pain due to QL spasm. AQLB use in the ED holds promise not only for acute back pain, specifically QL syndrome relief, but also for reducing opioid dependence, enabling early mobilisation, and preventing unnecessary hospital admissions, thereby improving patient outcomes while supporting healthcare efficiency. However, further studies are required to study the efficacy of the AQLB for the management of back pain in the ED.
